# Characterizing HIV-1 transmission by genetic cluster analysis among newly diagnosed patients in the China-Myanmar border region from 2020 to 2023

**DOI:** 10.1080/22221751.2024.2409319

**Published:** 2024-09-24

**Authors:** Huan Liu, Yichen Jin, Yuecheng Yang, Xing Duan, Yanfen Cao, Duo Shan, Chang Cai, Houlin Tang

**Affiliations:** a National Key Laboratory of Intelligent Tracking and Forecasting for Infectious Diseases, National Center for AIDS/STD Control and Prevention, Chinese Center for Disease Control and Prevention, Beijing, People’s Republic of China; b National Center for AIDS/STD Control and Prevention, Chinese Center for Disease Control and Prevention, Beijing, People’s Republic of China; c Department of STD/AIDS Prevention and Control, Dehong Prefecture Center for Disease Control and Prevention, Mangshi, People’s Republic of China

**Keywords:** HIV-1, molecular epidemiology, subtype, cluster analysis, cross-border

## Abstract

Cluster analysis of HIV sequence can provide insights into viral transmission patterns in border regions. This study aims to illuminate the HIV-1 subtype distribution and transmission dynamics among newly diagnosed individuals in Dehong prefecture, a region along the China-Myanmar border. Among 948 participants with *pol* gene sequences, 36 HIV-1 subtypes were identified, with URFs (18.8%, 178/948) being the dominant strain, followed by CRF01_AE (18.5%, 175/948) and CRF07_BC (10.9%, 103/948). Additionally, 287 sequences (30.3%, 287/948) were grouped into 91 clusters, 31 of which contained both Chinese and Burmese individuals. Multivariable logistic regression indicated that men who have sex with men (MSM), CD4 + cell count of 200∼499, and 500 cells/μl and above, and CRF01_AE were risk factors for entering the network. Through the Chord diagram, we found frequent transmission relationships among heterosexual China male group, especially those over 35 years of age. Additionally, the correlation between heterosexual Myanmar female group and heterosexual China male group among cross-risk groups deserved to be emphasized. Furthermore, the network exhibited a growing trend over time, with the largest active transmission cluster identified in Ruili county. In conclusion, the HIV-1 subtype landscape in Dehong has become increasingly complex, and the region has faced risks of transmission from both domestic and international sources. Targeted intervention strategies should be implemented for MSM, heterosexual Chinese middle-aged and elderly men, and heterosexual Burmese young adults to mitigate these risks. These findings provided evidence-based insights for local government to formulate coordinated transnational intervention approaches.

## Introduction

The HIV pandemic remains a major global public health problem and substantial HIV transmission continues to occur worldwide with population mobility. By analysing the genetic characteristics of HIV-1 strains in different regions, researchers can gain insight into the origins of HIV-1 strains and dynamics of HIV-1 transmission [1, 2]. Cross-border HIV-1 transmission can be facilitated by the mobility of high-risk populations, such as people who inject drugs (PWID) and sex workers [3, 4], changing local dynamics of transmission [5, 6]. Due to China's border-sharing with multiple countries, previous research has identified a higher prevalence of HIV-1 among transnational floating populations at Chinese border ports compared to the general population [4]. It is of great significance to emphasize the cross-border transmission of HIV-1 in border regions as a means of controlling the HIV epidemic in China.

Yunnan province, located in southern China and bordering Myanmar, Laos and Vietnam, is in close proximity to the “Golden Triangle,” a significant narcotic production area. Given its geographical position and high prevalence of HIV-1 infection, it has been regarded as a potential gateway for HIV-1 transmission between China and Southeast Asia [7–9]. By the end of 2021, the cumulative number of reported HIV-infected patients in Yunnan province had exceeded 170,000 [10]. In recent years, the HIV-1 epidemic has shown a downward trend in Yunnan province, with a decline in the number of newly reported HIV-infected individuals [11]. Dehong prefecture is a major region for trading in the Yunnan-Myanmar border area. In 1989, 146 cases of HIV-infected were identified in the PWID of Dehong, marking the first HIV-1 epidemic in China [12]. Since 2005, Dehong has implemented a comprehensive HIV prevention strategy of “Beforehand Prevention, Downward Shift of Governance Focus,” which was characterized by policy preference, whole-society participation, family-and-community-based support, professional technology, and joint implementation of multiple measures. By the end of 2019, Dehong had achieved a leading role in Yunnan province in meeting the “90-90-90” HIV prevention and treatment goals [13]. However, due to its special geographic location, there is still a long way to go in consolidating the achievements. In Dehong, the main mode of HIV-1 transmission has shifted from initial drug injection to heterosexual contact, associated with faster spread of multiple subtypes [14–16]. Previous studies demonstrated that Dehong has been a geographical hotspot for recombination of HIV-1 strains, with a high prevalence of both circulating recombinant forms (CRFs) and unique recombinant forms (URFs) [17, 18]. Consequently, the continuous monitoring of subtype changes would facilitate the understanding of HIV-1 transmission dynamics and epidemiological trends in the region. While the number of individuals newly diagnosed with HIV-1 in Dehong was decreasing, the proportion of Burmese nationals among these cases reached 60% and increased annually [19]. Currently, Burmese nationals voluntarily participate in HIV testing and receive antiretroviral therapy (ART) in China [20]. However, due to their high mobility, most can only receive one-time services, making it difficult to provide long-term treatment. Therefore, analysing the impact of Burmese nationals on the HIV-1 epidemic in Dehong prefecture is crucial for controlling HIV-1 transmission.

As molecular epidemiology advances, the molecular network, constructed based on genetic similarity among persons closely related by transmission, has emerged as a critical tool for HIV prevention strategies [21]. The cluster analysis can reveal potential factors promoting the HIV epidemic spread and identify emerging transmission clusters to tailor precise interventions [22, 23]. Nevertheless, research conducted in Dehong focused on simple distribution of HIV-1 subtype or transmission network within specific populations before 2019 [3, 14, 17, 24]. A comprehensive analysis of HIV-1 transmission dynamics across the entire population has been notably lacking.

In this study, we conducted a molecular epidemiological survey among Chinese and Burmese nationals in Dehong diagnosed between 2020 and 2023. Specifically, we aimed to characterize HIV-1 transmission among subjects and identify the drivers of the HIV-1 epidemic by integrating cluster analysis with epidemiological and laboratory data, providing a scientific basis for public health response in Dehong.

## Materials and methods

### Study participants and sample collection

For the cross-sectional study, we used the formula to calculate the sample size: $n=Z_{1-\alpha /2}^{2}\times p\times q/d^{2}$. We hypothesized a significance level of 0.05, resulting in a *Z*-value of 1.96. The *p* value represented the expected rate of clustering, i.e. 30% [25–27], then *q *= 1-*p* and *d *= 0.1*p*. Additionally, we factored in a 20% proportion of sequencing failure. Based on these parameters, the estimated sample size was 1076. Inclusion criteria for the participants included (1) confirmed HIV-positive between 2020 and 2023 in Dehong, (2) treatment-naïve during sampling, (3) provided signed informed consent.

Following confirmation of infection, healthcare staff would inform individuals of their HIV-positive status via phone, and schedule a time for face-to-face interview to collect demographic information (i.e. nationality, gender, age) and blood sample for sequencing. All pertinent information would be entered into China Information System for Disease Prevention and Control [28]. The informed consent was obtained from all subjects and/or their legal guardian(s). Encryption techniques were employed to ensure the confidentiality of data transfer and storage. All plasma samples were linked to epidemiological data using anonymous numerical codes, and no personal identification information was included in the data analysis. Additionally, we classified the heterosexual contact into heterosexual contact within spouse, commercial heterosexual contact (CHC, selling or buying sex), and nonmarital noncommercial heterosexual contact (NMNCHC, heterosexual contact with transient or casual partners without payment) to further understand transmission relationship [29]. This study was approved by the Institutional Review Board of the National Center for AIDS/STD Control and Prevention, Chinese Center for Disease Control and Prevention (No. X231018772).

### Amplification and sequencing of HIV-1 pol gene fragments

HIV RNA was extracted from plasma using a Roche fully automated nucleic acid extractor (MagNA Pure 2.0 Instrument) and its companion kit (MagNA Pure LC Total Nucleic Acid Isolation Kit) according to the manufacturer’s instructions. HIV-1 *pol* gene was amplified using reverse transcription-polymerase chain reaction (RT–PCR) and nested polymerase chain reaction (PCR). The primers, conditions and procedures for reverse transcription PCR were described in a previous study [30]. The positive PCR products were visualized in 1% agarose gel electrophoresis, and commercially sequenced by Biomed company (Beijing, China).

### Subtype identification and analysis

Sequences were assembled and edited in LaserGene 7.1 software. The assembled sequences were aligned with Bio-Edit 7.0 software and manually edited. HIV-1 reference sequences were selected and downloaded from the HIV databases of the Los Alamos National Laboratory (LANL) (http://www.hiv.lanl.gov). Then the processed sequences were submitted to China HIV Gene Sequences Database platform for subtype determination (https://nmdc.cn/hiv/tool/sequence). It is based on the preliminary determination of subtypes by HIV BLAST software, as well as the construction of a phylogenetic tree using FastTree software, which determines sample subtypes based on the topological relationship between the sample and the reference strain on the phylogenetic tree.

### Inference of molecular transmission network

The molecular transmission network was inferred based on pairwise genetic distance using HIV-TRACE (Transmission Cluster Engine) [31]. Using the Tamura-Nei 93 nucleotide substitution model (TN93) to calculate pairwise distance between all pairs of HIV-1 *pol* sequences. The ambiguous nucleotides of all sequences were less than 5%. The network data were visualized using the HIV-TRACE website (https://veg.github.io/hivtrace-viz/#). The degree indicates connectivity and represents the number of links or edges connecting to the other individuals in a molecular network. Clusters are defined as those connected components of the network comprising two or more nodes. In order to identify all possible associations between individuals, we linked nodes to each other if their pairwise genetic distance was up to 1.5% substitutions per site based on the recommended genetic distance threshold by the China CDC [32]. Additionally, in this study, a molecular network incorporating newly diagnosed HIV-infected patients from 2020 to 2021 served as the baseline network, and transmission clusters with a threshold of five or more newly reported cases in 2022 and 2023 were designated as active transmission clusters.

### Statistical analysis

The statistical analysis and visualization were executed using R version 4.2.3. Comparisons of categorical variables were carried out using the Chi-squared (*χ*^2^) test. Factors associated with clustering were evaluated by binomial logistic regression. All the variables of the univariable logistic regression were incorporated into the multivariable logistic regression model. The unadjusted *OR*, adjusted *OR*, and 95% *CI* were calculated. All tests were conducted with a two-tailed approach, and a *P*-value less than 0.05 was deemed statistically significant.

## Results

### Demographic characteristics of the study participants

**Figure 1.**
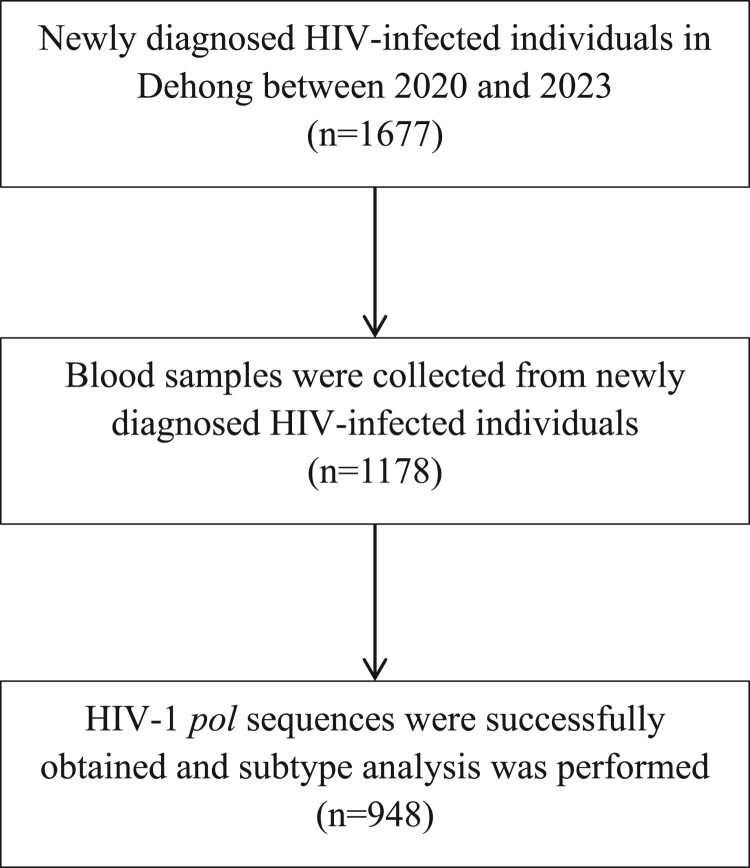
Flow chart shows the individuals meeting inclusion criteria.

A total of 1178 blood samples were collected from 1677 newly diagnosed HIV-infected individuals in Dehong between 2020 and 2023. Of these samples, 948 HIV-1 *pol* gene sequences were successfully amplified (Figure 1). Between 1677 newly diagnosed patients and 1178 individuals with blood samples, the most demographic characteristics showed no significant differences except for nationality and ethnicity (Table S1). The proportion of Burmese nationals and Dai individuals sampled was relatively low.

Of the 948 subjects with *pol* sequences, 543 (57.3%) were from China, of which 485 (89.3%) were local residents and 58 (10.7%) were from other cities in China. Additionally, 405 (42.7%) were from Myanmar. Furthermore, 597 (63.0%) were male, 412 (43.5%) were married, 344 (36.3%) were Han ethnicity, and 807 (76.2%) were with middle school education or below. Heterosexual contact accounted for 82.4% (781/948), of which 73.6% (575/781) were NMNCHC, 13.4% (105/781) were heterosexual contact within spouse, and 11.5% (90/781) were CHC. Among the 935 subjects with CD_4_+ cell count data, 481 (51.4%) presented with counts 200∼499 cells/μl.

### The distribution of HIV-1 subtypes

Among the 948 *pol* sequences, 36 HIV-1 strain subtypes were identified, including subtype B, subtype C, 33 CRFs and discrete URFs. URFs strains were the predominant subtypes, accounting for 18.8% (178/948), followed by CRF01_AE (18.5%, 175/948), CRF07_BC (10.9%, 103/948), subtype B (10.1%, 96/948), subtype C (9.5%, 90/948), CRF08_BC (8.6%, 82/948), and other subtypes (23.6%, 224/948).

**Figure 2.**
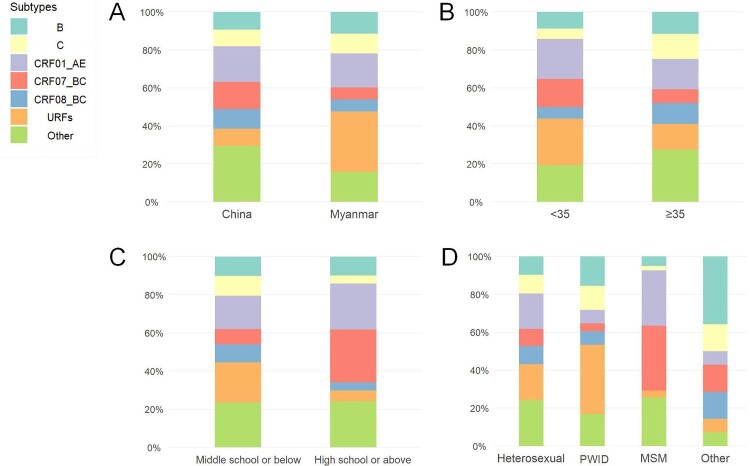
Distribution of subtypes by different characteristics. A. subtypes distribution by nationality. B. subtypes distribution by age at diagnosis. C. subtypes distribution by education level. D. subtypes distribution by mode of transmission.

The distribution of HIV-1 subtypes varied by nationality, age, education level, and mode of transmission (Figure 2). In Burmese nationals, 31.9% (129/405) of sequences were classified as URFs, and 18.0% (73/405) as CRF01_AE. Whereas CRF01_AE (18.8%, 102/543) was the dominant subtype among Chinese individuals, followed by CRF07_BC (14.4%, 78/543). Additionally, a high prevalence of URFs was observed in individuals under the age of 35, with a low level of literacy, and those who were PWID. Furthermore, CRF07_BC was more prevalent than CRF01_AE among the subpopulation with an education level of high school or above (27.7%, 39/141 vs. 24.1%, 34/141) and those infected through homosexual contact (34.1%, 28/82 vs. 29.3%, 24/82) (Table S2).

### Characteristics of transmission networks

**Figure 3.**
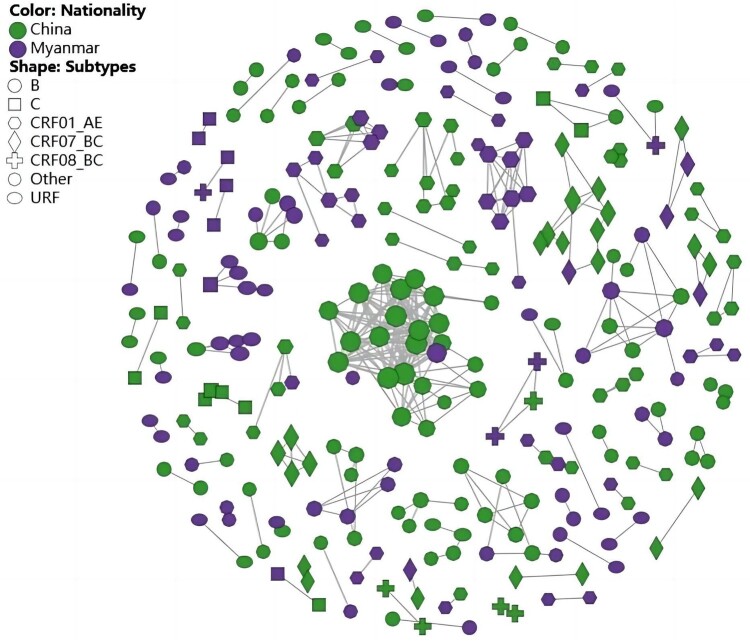
Molecular transmission network analysis of HIV-1 among newly diagnosed patients in Dehong from 2020 to 2023.

Under the threshold of 1.5% genetic distance, 287 sequences (30.3%, 287/948) were grouped into 91 clusters with a total of 374 links, ranging in size from 2 to 25 sequences (Figure 3). A total of 21 HIV-1 subtypes were enrolled in the network, dominated by CRF01_AE (27.5%, 79/287) and URFs (16.4%, 47/287) (Table 1).

**Table 1.** Factors associated with clustering in the transmission network.

Characteristics	Total	Clustered in network, n(%)	Univariable analysis		Multivariable analysis	
			*OR* (95% *CI)*	*P-*value	a*OR* (95% *CI)*	*P-*value
Nationality						
China	543	175 (32.2)	Ref.		Ref.	
Myanmar	405	112 (27.7)	0.804 (0.605∼1.065)	0.130	0.971 (0.670∼1.407)	0.878
Gender						** **
Male	597	199 (33.3)	1.494 (1.115∼2.014)	0.008	1.387 (0.981∼1.967)	0.065
Female	351	88 (25.1)	Ref.		Ref.	
Age at diagnosis (years)						
<35	459	158 (34.4)	Ref.		Ref.	
≥35	489	129 (26.4)	0.683 (0.516∼0.901)	0.007	0.719 (0.496∼1.041)	0.081
Marital status						
Married	412	124 (30.1)	Ref.		Ref.	
Unmarried	321	108 (33.6)	1.178 (0.861∼1.610)	0.306	0.744 (0.494∼1.116)	0.155
Divorced or widowed	215	55 (25.6)	0.798 (0.548∼1.154)	0.235	0.875 (0.584∼1.303)	0.513
Ethnicity						
Han	344	117 (34.0)	Ref.		Ref.	
Dai	216	74 (34.3)	1.011 (0.705∼1.446)	0.952	1.067 (0.715∼1.591)	0.749
Jingpo	269	53 (19.7)	0.476 (0.326∼0.689)	<0.001	0.519 (0.332∼0.803)	**0**.**004**
Other	119	43 (36.1)	1.098 (0.707∼1.691)	0.674	1.107 (0.674∼1.807)	0.686
Education level						
Middle school or below	807	227 (28.1)	Ref.		Ref.	
High school or above	141	60 (42.6)	1.893 (1.307∼2.729)	<0.001	1.362 (0.862∼2.140)	0.182
Mode of transmission						
Heterosexual	781	229 (29.3)	Ref.		Ref.	
PWID	71	12 (16.9)	0.490 (0.247∼0.898)	0.029	0.503 (0.239∼0.987)	**0**.**055**
MSM	82	44 (53.7)	2.791 (1.762∼4.440)	<0.001	1.804 (1.026∼3.190)	**0**.**041**
Other	14	2 (14.3)	0.402 (0.062∼1.488)	0.235	0.661 (0.097∼2.701)	0.608
CD4+ cell count (cells/μl)						
<200	243	53 (21.8)	Ref.		Ref.	
200∼499	481	151 (31.4)	1.640 (1.150∼2.366)	0.007	1.498 (1.025∼2.210)	**0**.**039**
≥500	211	77 (36.5)	2.060 (1.365∼3.127)	<0.001	1.996 (1.282∼3.126)	**0**.**002**
Unknown	13	6 (46.2)	3.073 (0.953∼9.634)	0.052	2.492 (0.728∼8.309)	0.134
Subtype						
B	96	17 (17.7)	0.576 (0.287∼1.129)	0.113	0.960 (0.457∼1.988)	0.913
C	90	16 (17.8)	0.579 (0.285∼1.147)	0.122	0.962 (0.448∼2.030)	0.920
URF	178	47 (26.4)	0.961 (0.558∼1.673)	0.887	1.852 (0.993∼3.519)	0.056
CRF01_AE	175	79 (45.1)	2.204 (1.313∼3.771)	0.003	2.888 (1.649∼5.175)	**<0**.**001**
CRF07_BC	103	28 (27.2)	Ref.		Ref.	
CRF08_BC	82	9 (11.0)	0.330 (0.139∼0.723)	0.008	0.522 (0.210∼1.206)	0.140
Other	224	91 (40.6)	1.833 (1.111∼3.085)	0.020	2.648 (1.526∼4.711)	**<0**.**001**

Notes: MSM, men who have sex with men; PWID, people who inject drugs, Unknown, data are not available; OR, unadjusted odds ratio; aOR, adjusted odds ratio; CI, confidence interval; Ref., reference.

The molecular network exhibited a growing trend over time. Within the network, 167 (58.2%, 167/287) of HIV-infected individuals diagnosed in 2022 or 2023 were grouped into 63 clusters, of which 31 clusters contained only participants diagnosed in 2022 or 2023. Additionally, 161 (56.1%, 161/287) local residents, 14 (4.9%, 14/287) residents from other cities in China and 112 Burmese nationals (39.0%, 112/287) were included in network. Notably, 32.9% (123/374) of the links were identified between local residents and non-local residents, and 42.0% (47/112) of Burmese nationals were linked with Chinese. Among the 91 clusters, 37 and 23 clusters involved Chinese and Burmese nationals only, respectively, and other 31 clusters involved mix nationality groups. Furthermore, heterosexual contact accounted for 79.7% (229/287), followed by 15.3% (44/287) who were men who have sex with men (MSM), and 13 clusters involved both MSM and heterosexual individuals.

### Factors associated with clustering

To explore features among the clustering and non-clustering individuals, we investigated differences between the two groups (Table 1). Univariable analysis showed that the risk factors for clustering were gender, age, ethnicity, education level, mode of transmission, CD4 + cell count and subtype. In the multivariable logistic regression, participants were more likely to cluster if they were MSM compared to heterosexuals (*aOR *= 1.804, 95% *CI*: 1.026∼3.190), had a CD4 + cell count of 200∼499 cells/μl (*aOR *= 1.498, 95% *CI*: 1.025∼2.210) or ≥500 cells/μl (*aOR *= 1.996, 95% *CI*: 1.282∼3.126) compared to less than 200 cells/μl, or CRF01_AE (*aOR *= 2.888, 95% *CI*: 1.649∼5.175) compared to CRF07_BC. Participants were less likely to cluster if they were Jingpo ethnicity (a*OR *= 0.519, 95% *CI*: 0.332∼0.803), or PWID (*aOR *= 0.503, 95% *CI*: 0.239∼0.987), with the latter showed marginal significance.

### Risk group characteristics in the network

**Figure 4.**
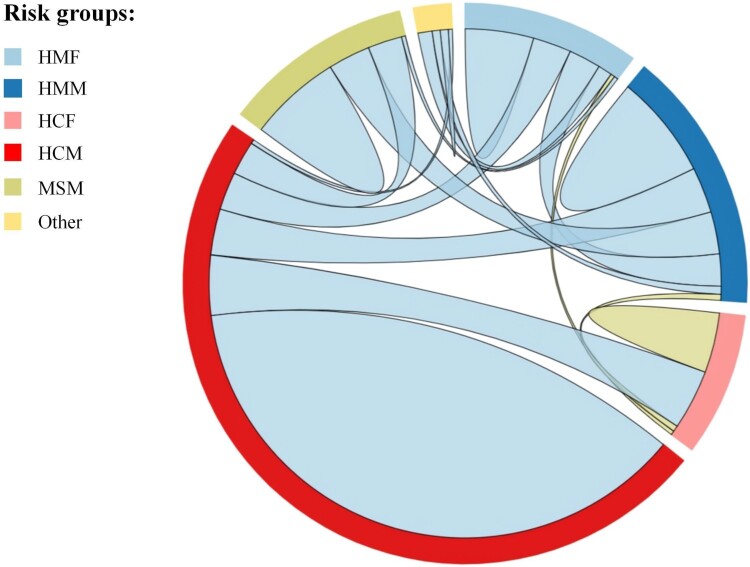
Linkage analysis of different risk groups in the network. Abbreviations: HMF: heterosexual-Myanmar-female,HMM:heterosexual-Myanmar-male, HCF: heterosexual-China-female, HCM: heterosexual-China-male, MSM: men who have sex with men.

The links connected to various risk groups in the network reflect the relationship and risk among these groups. Hence, we labelled risk groups by “mode of transmission-nationality-gender,” which were classified heterosexual-China-male (HCM), heterosexual-China-female (HCF), heterosexual-Myanmar-male (HMM), heterosexual-Myanmar-female (HMF), MSM and others. We drew a Chord diagram based on the links among various risk groups (Figure 4). Among all 374 links, the correlation between HCM in the same risk groups was the highest at 38.8% (145/374), with a high proportion of associations between individuals aged 35 years and older (91.0%, 132/145). Additionally, most risk groups, including HCM, HMM, MSM and others, had a higher proportion of links to the same risk group than to other risk group. However, the links between HMF and HCM among cross-risk groups exceeded the links between HMF in the same groups, as well as the links between HCF and HCM among cross-risk groups exceeded the links between HCF in the same groups.

### Characterizing network dynamic changes

**Figure 5.**
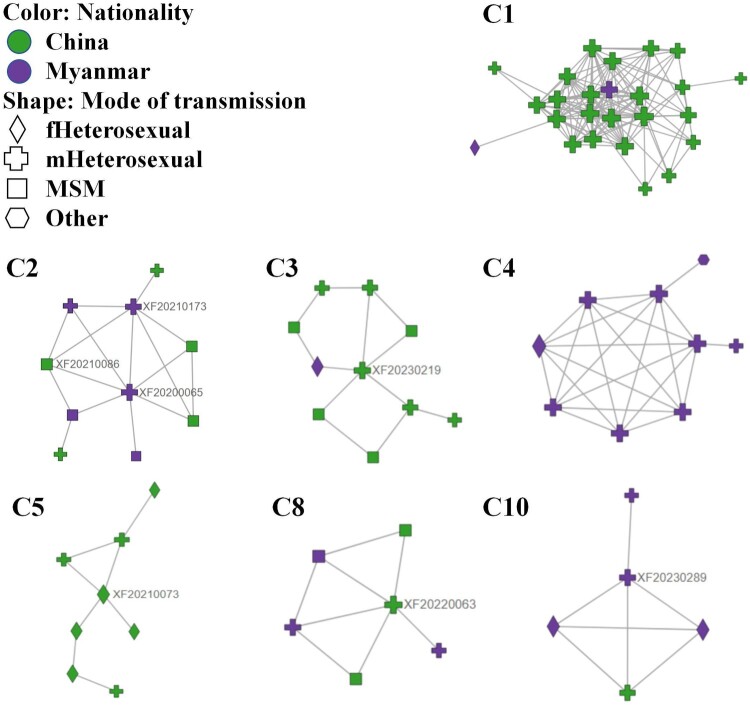
Analysis of active transmission clusters within the transmission network. Abbreviations: fHeterosexual, heterosexual female; mHeterosexual, heterosexual male.

Seven active transmission clusters with 73 individuals were identified, accounted for 25.4% (73/287) of participants entering the network, including C1∼C5, C8 and C10 (Table 2 and Figure 5). A total of 5 subtypes were identified in the participants, namely, CRF64_BC, CRF55_01B, CRF07_BC, CRF01_AE, and subtype B.

**Table 2.** Analysis of active transmission clusters among newly diagnosed HIV-infected patients.

				Characteristics within the active clusters, n (%)					
Name of cluster	Subtype	Additional cases in 2022	Additional cases in 2023	Chinese	Male	Married	Middle school or below	≥35 years old	Heterosexual contact
C1(n = 25)	CRF64_BC	4	20	23 (92.0)	24 (96.0)	15 (60.0)	22 (88.0)	25 (100.0)	25 (100.0)
C2(n = 10)	CRF55_01B	3	3	5 (50.0)	10 (100.0)	2 (20.0)	6 (60.0)	2 (20.0)	5 (50.0)
C3(n = 10)	CRF07_BC	2	6	9 (90.0)	9 (90.0)	2 (20.0)	1 (10.0)	2 (20.0)	6 (60.0)
C4(n = 9)	CRF01_AE	0	8	0	8 (88.9)	0	8 (88.9)	0	8 (88.9)
C5(n = 8)	CRF01_AE	4	1	8 (100.0)	3 (37.5)	5 (62.5)	8 (100.0)	8 (100.0)	8 (100.0)
C8(n = 6)	B	3	3	3 (50.0)	6 (100.0)	0	4 (66.7)	1 (16.7)	3 (50.0)
C10(n = 5)	B	2	3	1 (20.0)	3 (60.0)	5 (100.0)	5 (100.0)	3 (60.0)	5 (100.0)

The largest active cluster, C1, comprised 25 individuals. Of these, 21 were Chinese men aged 45 years or older (84.0%), and 16 were infected through CHC. Additionally, 24 individuals were concentrated in Ruili county, indicating the possibility of regional clustering. Clusters C2, C3, C8, and C10 were composed of mixed nationality individuals. Among them, 41.9% (13/31) were Burmese nationals, most of whom had a middle school education or below (84.6%, 11/13) and a history of nonmarital noncommercial heterosexual behaviour (61.5%, 8/13). Meanwhile, 58.1% of the subjects were Chinese, with a high school education (72.2%, 13/18) and a history of homosexual behaviour (55.6%, 10/18). Additionally, C2∼C3, and C8 had both heterosexual and homosexual contact. Cluster C4 was entirely composed of Burmese nationals, mostly unmarried, with a history of nonmarital noncommercial heterosexual behaviour (88.9%, 8/9), and aged 17–32. Cluster C5, on the other hand, consisted solely of Chinese, mostly married, aged 38–62, and two individuals had HIV-positive spouses.

Furthermore, within clusters C2, C3, C5, and C8, subjects identified as XF20200065, XF20210086, XF20230219, XF20210073, and XF20220063 played pivotal roles. Importantly, each of these subjects had initial CD4 + cell counts of less than 200 cells/μl. Additionally, two participants, identified as XF20210173 in C2 and XF20230289 in C10, were Burmese nationals with a middle school education or below (Table S3).

## Discussion

Cross-border individual mobility is commonly recognized as a pivotal factor in the inter-regional spread of HIV epidemics [6, 17]. In this study, we conducted a detailed molecular epidemiological study involving subtype distribution, network characteristics, risk group, and active cluster analyses. We not only revealed the existence of cross-border HIV-1 transmission based on genetic evidence, but also pinpointed the key populations driving HIV-1 epidemics in Dehong.

We found that the subtypes in China-Myanmar border were diverse, and much more complex than in most regions [25, 33]. In Dehong, the distribution of HIV-1 subtypes was dominated by URFs, followed by CRF01_AE and CRF07_BC. Compared to the local surveillance results of 2017—2019 [24], a significant increase was observed in the proportion of URFs and CRFs, indicating more complex recombinants of the HIV-1 subtypes in Dehong. Indeed, the distribution of subtypes varies from region to region [34]. There was a significant difference in the composition of HIV-1 subtypes between Chinese and Burmese strains, with a higher prevalence of CRFs and URFs among Chinese and Burmese nationals, respectively. The result was consistent with a similar survey conducted in Baoshan, another China-Myanmar border region [25]. These findings further showed the unique subtype composition and wide range of transmission sources in Dehong. It is necessary to conduct continuous subtype monitoring of HIV-infected patients in Dehong and implement targeted interventions to control HIV-1 prevalence and transmission.

With the advancement of the economy and transportation, inter-regional transmission of HIV has become an increasingly prevalent issue. The transmission of HIV between individuals from different regions significantly enhanced the opportunities for viral recombination, leading to an increased complexity of genetypes [5, 6]. Our study found approximately one-third of the transmission links occurred between local and non-local residents. Indeed, the Burmese nationals constituted the majority among non-local residents. This further emphasized the necessity to consider the dynamics of HIV transmission among migrants on local contexts, especially Burmese nationals.

Notably, our study provided direct genetic evidence of transmission linkages between Chinese and Burmese nationals. The increasing number of recombinant subtypes suggested extensive cross-over transmission among participants in the region [35, 36]. We found 31 clusters containing both Chinese and Burmese nationals. Moreover, 42% of Burmese nationals had a transmission linkage with Chinese. We also observed relatively frequent transmission relationships between heterosexual Burmese female and heterosexual Chinese male. Furthermore, active cluster analysis revealed that heterosexual Burmese young male adults may be the driving force behind the growth of clusters. Consistent with previous research highlighting the significant role of young adults in Myanmar in the transmission of HIV-1 [14]. The majority of Burmese nationals in Dehong who are sexually active, have migrated to China for work, medical care, or marriage, exhibit frequent entry and exit behaviours and possess a low level of education [19]. These findings further validated the potential risk of cross-border HIV-1 transmission along the China-Myanmar border [3, 14]. Notably, transborder transmission of HIV posed a great challenge to ART. Firstly, frequent cross-transmission increased the genetic diversity of HIV-1, which can lead to increased pressure for drug selection and the the potential for drug resistance [37]. Secondly, cross-border transmission accelerated the spread of drug-resistant strains between different regions. Therefore, it is imperative for local government to prioritize the prevention of cross-border HIV transmission, explore effective management and treatment strategies for Burmese nationals, and establish a joint HIV-infection prevention and control framework in collaboration with the Myanmar government.

Our study further confirms that heterosexual contact remained the primary mode of transmission in Dehong, with NMNCHC accounting for 73.6%. Regional studies also noticed that NMNCHC accounted for an increasingly high proportion of heterosexual contact, which indicated its growing significance [29, 38]. Individuals infected with HIV through this mode were difficult to identify in the general population. This invisibility necessitates innovative strategies to reach and support these individuals effectively. Additionally, we found a strong transmission relationship among middle-aged and elderly people, with 91% of links among heterosexual Chinese male occurring between middle-aged and elderly people. The elderly population is characterized by a lack of awareness regarding HIV and often engage in unprotected sexual behaviours driven by emotional needs, significantly increasing their susceptibility to HIV infection [39]. For this concealed high-risk population, efficacious intervention strategies, encompassing education, accessibility of pre-exposure prophylaxis (PrEP), and behavioural interventions, must be continuously implemented. To our knowledge, empirical evidence for the deployment of PrEP is currently scarce within the region, indicating the significant potential for future application of PrEP in this context.

The proportion of MSM in Dehong has exhibited an upward trend over time [40]. Compared with heterosexual contact, MSM were more likely to belong to clusters. Contrasting the MSM survey in Dehong from 2010 to 2019 [41], we observed a higher prevalence of CRF07_BC as opposed to CRF01_AE, which was consistent with adjacent areas [42, 43]. The CRF07_BC, exhibiting higher propagation dynamics than CRF01_AE, has contributed to the highest number of HIV infection and was the most predominant strain spreading interprovincially in China [44–46]. The MSM population typically exhibits high levels of mobility and social activities, potentially leading to a more aggregated and closed transmission scenario [47]. Additionally, we found that MSM in the network were also linked to heterosexual male and female. This result may be explained by two potential explanations. First, these heterosexual males may have chosen not to disclose a history of homosexual behaviour due to stigma and discrimination. Second, these MSM may have engaged in both heterosexual and homosexual behaviours, consequently, the key role of this “bridge population” in HIV-1 transmission should be emphasized [22].

Additionally, we found PWID were less likely to have clustered infections than heterosexual populations. The finding differed from a study conducted in Dehong between 2009 and 2017 [14]. This indicated that HIV transmission among PWID has been effectively reduced. A study showed that the proportion of newly diagnosed HIV individuals in China infected through drug-injection decreased annually, from 10.1% in 2012 to 1.2% in 2019 [48]. The latest report showed that the proportion was only 0.3% in 2023 [49]. This may be attributed to strict drug control policies (i.e. drug use and trafficking), effective harm reduction interventions (i.e. needle change and opioid substitution therapy), and improved local health service accessibility.

Not all clusters growth is equivalent, consequently, targeting active clusters and core populations with measures to curb risk behaviours are probable to reduce HIV transmission, thereby consolidating the achievements of local HIV-1 prevention. We identified a total of 7 active clusters, the largest of which was observed in Ruili county, suggesting a possible outbreak in that region. Ruili county is a major foreign trade port in the southwest of China, with a considerable domestic and foreign mobile population [50]. Additionally, the cluster had both NMNCHC and CHC, indicating complexity of transmission. Post the COVID-19 pandemic, as trade between China and Myanmar resumes progressively and population mobility intensifies, it is imperative to highlight the potential importation of HIV from Myanmar. Moreover, we found lower CD4 + cell counts in nodes at key positions of active clusters, which indicated that some delayed diagnosed individuals were still transmitting HIV, hence continue expanding HIV testing was also necessary to curb HIV spread for local government.

This study also has several limitations. First, the relatively low sampling rate among Burmese nationals might have resulted in an underestimation of the transmission risk. Second, the molecular transmission network could provide an inferred transmission relationship based on a close genetic distance, while true transmission relationships need to be confirmed through epidemiological investigations. Nonetheless, the common characteristics exhibited by individuals within clusters offer valuable insights that can inform future epidemiological research.

Taken together, we presented a comprehensive molecular epidemiology investigation among newly diagnosed patients between 2020 and 2023 in the China-Myanmar border region. In Dehong, the HIV-1 strains exhibited a complex distribution of subtypes, exposing the region to bivariate transmission risks from both domestic and international sources. These data highlight distinct spatial and demographic clustering in this region, with a targeted focus on MSM, heterosexual Chinese middle-aged and elderly male, and heterosexual Burmese young adults for intervention strategies. These findings provide evidence-based insights for local government to formulate coordinated transnational intervention approaches.

## Supplementary Material

Supplemental_material-clean.docx
