# Supplementary material for: Characterizing HIV-1 transmission by genetic cluster analysis among newly diagnosed patients in the China-Myanmar border region from 2020 to 2023
Source: Emerg Microbes Infect. 2024 Sep 24;13(1):2409319. doi: 10.1080/22221751.2024.2409319 (PMC11443545; doi:10.1080/22221751.2024.2409319)
Supplement: Supplemental_material-clean.docx [file TEMI_A_2409319_SM4720.docx]

**Characterizing HIV-1 transmission by genetic cluster analysis among newly diagnosed patients in the China-Myanmar border region from 2020 to 2023**

**Supplementary Information**

**Table S1.** Sociodemographic characteristics of newly diagnosed HIV-infected patients and those infected with access to plasma samples in Dehong prefecture from 2020 to 2023.

| Characteristics | Newly diagnosed patients  (n=1677) | Plasma samples  (n=1178) | *χ*^2^ | *P*-value |
| --- | --- | --- | --- | --- |
| Nationality |  |  | 11.859 | **<0.001** |
| China | 793 | 635 |  |  |
| Myanmar | 884 | 543 |  |  |
| Gender |  |  | 1.260 | 0.262 |
| Male | 1016 | 739 |  |  |
| Female | 661 | 439 |  |  |
| Age at diagnosis |  |  | 0.102 | 0.749 |
| <35 | 825 | 571 |  |  |
| ≥35 | 852 | 607 |  |  |
| Marital status |  |  | 2.861 | 0.414 |
| Married | 773 | 530 |  |  |
| Single | 571 | 401 |  |  |
| Divorced or widowed | 330 | 247 |  |  |
| Unknown | 3 | 0 |  |  |
| Ethnicity |  |  | 12.590 | **0.006** |
| Han | 538 | 406 |  |  |
| Dai | 508 | 289 |  |  |
| Jingpo | 436 | 348 |  |  |
| Other | 195 | 135 |  |  |
| Education level |  |  | 1.668 | 0.197 |
| Middle school or below | 1471 | 1013 |  |  |
| High school or above | 206 | 165 |  |  |
| Mode of transmission |  |  | 4.177 | 0.243 |
| Heterosexual | 1439 | 980 |  |  |
| PWID | 112 | 91 |  |  |
| MSM | 103 | 91 |  |  |
| Other | 23 | 16 |  |  |
| CD4+ cell count (cells/μl） |  |  | 0.505 | 0.777 |
| <200 | 355 | 275 |  |  |
| 200~ | 710 | 583 |  |  |
| ≥500 | 383 | 298 |  |  |
| Unknown | 229 | 22 |  |  |

Abbreviations: MSM, men who have sex with men; PWID, people who inject drugs, Unknown, data are not available.

**Table S2.** Distribution of HIV-1 subtypes among study participants

| Characteristics | Total  （n=948） | Subtypes | | | | | | |
| --- | --- | --- | --- | --- | --- | --- | --- | --- |
|  |  | B  (n=96) | C  (n=90) | URFs  (n=178) | CRF01_AE  (n=175) | CRF07_BC  (n=103) | CRF08_BC  (n=82) | Other^a^  (n=224) |
| Nationality |  |  |  |  |  |  |  |  |
| China | 543 | 50 | 48 | 49 | 102 | 78 | 56 | 160 |
| Myanmar | 405 | 46 | 42 | 129 | 73 | 25 | 26 | 64 |
| Age group |  |  |  |  |  |  |  |  |
| <35 | 459 | 40 | 25 | 112 | 97 | 68 | 28 | 89 |
| ≥35 | 489 | 56 | 65 | 66 | 78 | 35 | 54 | 135 |
| Education level |  |  |  |  |  |  |  |  |
| Middle school or below | 807 | 82 | 84 | 170 | 141 | 64 | 76 | 190 |
| High school or above | 141 | 14 | 6 | 8 | 34 | 39 | 6 | 34 |
| Transmission route |  |  |  |  |  |  |  |  |
| Heterosexual | 781 | 76 | 77 | 148 | 145 | 70 | 75 | 190 |
| PWID | 71 | 11 | 9 | 26 | 5 | 3 | 5 | 12 |
| MSM | 82 | 4 | 2 | 3 | 24 | 28 | 0 | 21 |
| Other | 14 | 5 | 2 | 1 | 1 | 2 | 2 | 1 |

Notes: ^a^Other included CRF62_BC、CRF64_BC、CRF55_01B、CRF65_cpx、CRF96_cpx、CRF87_cpx、CRF88_BC、CRF15_01B、CRF110_BC、CRF103_01B、CRF118_BC、CRF57_BC、CRF106_cpx、CRF115_01C、CRF100_01C、CRF102_0107、CRF105_0108、CRF113_0107、CRF114_0155、CRF48_01B、CRF52_01B、CRF58_01B、CRF79_0107、CRF80_0107、CRF85_BC、CRF33_01B、CRF53_01B、CRF59_01B、CRF67_01B and CRF68_01B.

**Table S3**. Sociodemographic characteristics of key nodes within active transmission clusters in Dehong profecture from 2020 to 2023.

| Cluster | Nodes | Nationality | Gender | Age^a^ | Marital status | Education level^b^ | Mode of transmission | CD_4_+ cell count |
| --- | --- | --- | --- | --- | --- | --- | --- | --- |
| C2 | XF20200065 | Myanmar | Male | 21 | Unmarried | Low | Heterosexual | <200 |
|  | XF20210086 | China | Male | 30 | Married | High | MSM | <200 |
|  | XF20210173 | Myanmar | Male | 29 | Unmarried | Low | Heterosexual | 200~499 |
| C3 | XF20230219 | China | Male | 34 | Unmarried | High | Heterosexual | <200 |
| C5 | XF20210073 | China | Female | 52 | Married | Low | Heterosexual | <200 |
| C8 | XF20220063 | China | Male | 46 | Divorced/Widower | High | Heterosexual | <200 |
| C10 | XF20230289 | Myanmar | Male | 28 | Married | Low | Heterosexual | ≥500 |

Notes: Age^a^: age at diagnosis. Education level^b^: Low, middle school or below; High, high school or above.
